# Identifying phenotype-associated subpopulations through LP_SGL

**DOI:** 10.1093/bib/bbad424

**Published:** 2023-11-25

**Authors:** Juntao Li, Hongmei Zhang, Bingyu Mu, Hongliang Zuo, Kanglei Zhou

**Affiliations:** College of Mathematics and Information Science, Henan Normal University, 46 Jianshe East Road, 453007, Xinxiang, China; College of Mathematics and Information Science, Henan Normal University, 46 Jianshe East Road, 453007, Xinxiang, China; College of Arts and Design, Zhengzhou University of Light Industry, No. 5 Dongfeng Road, 450000, Zhengzhou, China; College of Mathematics and Information Science, Henan Normal University, 46 Jianshe East Road, 453007, Xinxiang, China; School of Computer Science and Engneering, Beihang University, 37 Xueyuan Road, Haidian District, 100191, Beijing, China

**Keywords:** data integration, cell–cell interaction, cell subpopulation, biological analysis

## Abstract

Single-cell RNA sequencing (scRNA-seq) enables the resolution of cellular heterogeneity in diseases and facilitates the identification of novel cell types and subtypes. However, the grouping effects caused by cell–cell interactions are often overlooked in the development of tools for identifying subpopulations. We proposed LP_SGL which incorporates cell group structure to identify phenotype-associated subpopulations by integrating scRNA-seq, bulk expression and bulk phenotype data. Cell groups from scRNA-seq data were obtained by the Leiden algorithm, which facilitates the identification of subpopulations and improves model robustness. LP_SGL identified a higher percentage of cancer cells, T cells and tumor-associated cells than Scissor and scAB on lung adenocarcinoma diagnosis, melanoma drug response and liver cancer survival datasets, respectively. Biological analysis on three original datasets and four independent external validation sets demonstrated that the signaling genes of this cell subset can predict cancer, immunotherapy and survival.

## INTRODUCTION

Human tumors are complex ecosystems composed of multiple cell types [1]. Fortunately, the increasing availability of omics data has provided important support for unraveling the complex features of tumors [2, 3]. Bulk data represent the average measurement of the entire tissue, while single-cell RNA sequencing (scRNA-seq) offers advantages in identifying cell types and therapeutic targets by revealing intratumoral heterogeneity [1, 4, 5]. Cell types are typically annotated by marker genes [6], but determining the role of specific cells in driving sample phenotypes remains a challenge. Although scRNA-seq data can provide high-resolution cell type information, it frequently lacks adequate sample phenotypes and clinical information due to its high cost [1]. Conversely, publicly available databases such as TCGA [7] contain a large amount of bulk data with sample phenotypes and clinical information.

Integrating bulk and scRNA-seq data effectively leverages the benefits of both phenotype and single-cell information simultaneously. Using scRNA-seq data, significant genes were selected as features to build a predictive breast cancer prognosis model with bulk data [8]. To identify subpopulations associated with sample phenotype, Scissor was developed with a sparse regression model [9]. In addition, scAB was developed to detect clinically significant multiresolution cell states using a knowledge- and graph-guided matrix factorization method [10]. As biological processes depend on complex interactions among different cells, we contend that incorporating cell group structure into the model will facilitate the identification of subpopulations associated with the phenotype. The implementation of Scissor and scAB relies on a correlation matrix, which comprises Pearson correlation coefficients of shared genes from bulk and scRNA-seq data. The screening of differentially expressed genes (DEGs) may potentially influence the performance of these methods. Thus, integrating the cell group structure into the model is likely to bolster its robustness.

Feature grouping has been considered in previous studies. Group lasso (GL) method was introduced to select features at the group level while performing regression [11]. To achieve intragroup sparsity, the sparse group lasso (SGL) was formulated for applications in linear regression, logistic regression and Cox regression [12]. A fundamental requirement for successfully applying SGL to bioinformatics is to group features beforehand. Although weighted gene co-expression network analysis (WGCNA) has been successfully applied to gene grouping of cancer bulk data [13, 14], it is not readily applicable to scRNA-seq data due to a large number of genes and cells. Therefore, identifying biologically meaningful group structures for scRNA-seq data is a challenging problem. Fortunately, community clustering algorithms such as Louvain [15] and Leiden [16] present promising avenues to solve this problem.

Inspired by the similarity between community connectivity and cell–cell interactions, we considered the cell communities obtained by the Leiden algorithm on scRNA-seq data as cell groups. We then proposed LP_SGL which incorporates cell group structure to identify phenotype-associated subpopulations by integrating scRNA-seq, bulk expression and bulk phenotype data. The experimental results showed that LP_SGL outperformed Scissor and scAB on datasets related to lung adenocarcinoma (LUAD) diagnosis, melanoma drug response and liver cancer survival. The robustness of the three methods was tested on seven datasets, including six incomplete datasets obtained under different threshold conditions. The subpopulation identification performance of LP_SGL remained almost unchanged, while the latter two methods showed significant fluctuations. Furthermore, the biological analysis confirmed the effectiveness of the proposed method.

## MATERIALS AND METHODS

### The structure of LP_SGL

**Figure 1:**
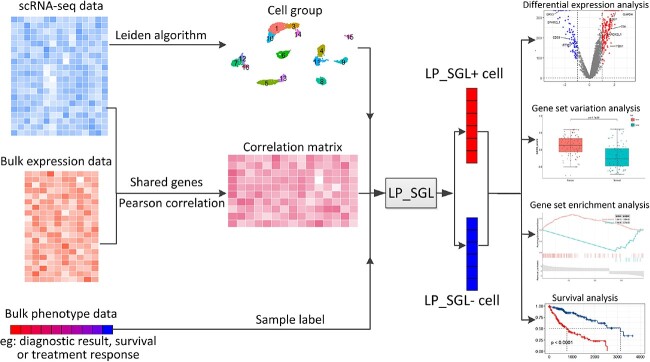
The workflow of LP_SGL.

LP_SGL is a specialized SGL [12] model that integrates scRNA-seq, bulk expression and bulk phenotype data. The model calculates the Pearson correlation coefficients between samples and cells by sharing genes and integrates scRNA-seq and bulk expression data into a correlation matrix. The letter ‘L’ indicates the use of the Leiden algorithm to obtain the cellular community structure from the scRNA-seq data. The letter ‘P’ represents the use of phenotype information to construct sample labels. The LP_SGL workflow was presented in Figure 1.

The Leiden algorithm [16] partitions nodes in a graph based on their similarity, which is analogous to each cell group representing a collection of cells with similar characteristics or functions. Therefore, it is reasonable to consider the cell communities obtained by the Leiden algorithm on scRNA-seq data as cell groups. Before executing the Leiden algorithm, the shared nearest neighbor graph was first constructed. Then, cells were divided into communities by maximizing the following modularity score: 


(1) \begin{align*}& Q=\frac{1}{2m}\sum_{i,j}{(A_{ij}-\gamma\frac{k_{i}k_{j}}{2m})}\delta(c_{i},c_{j}),\end{align*}

where $m$ stands for the total number of edges in the graph, $A_{ij}$ represents the weight of the edge between cell $i$ and $j$, $\gamma>0$ is a resolution parameter, $k_{i}$ and $k_{j}$ are the degrees of cell $i$ and cell $j$, respectively. $c_{i}$ denotes the community to which cell $i$ is assigned, the $\delta $ function is 1 if $c_{i}=c_{j}$ and 0 otherwise. The Leiden algorithm utilizes an iterative approach to enhance the initial partition by exchanging cells between communities to maximize the modularity score. This process continues until no further improvement is achievable. The algorithm was implemented through the R package ‘leidenAlg’.

Let $s$ be the number of the obtained cell groups, and $p_{l}$ be the number of cells in the $l$th group. Let $x_{i}$ be the $i$th row vector from the correlation matrix, and $x_{i}^{(l)}$ be its subvector corresponding to the $l$th group. LP_SGL can be described as 


(2) \begin{align*}& \min_{\beta}\frac{1}{n}l(\beta)+\lambda\left\{(1-\alpha)\sum_{l=1}^{s}{\sqrt{p_{l}}}||\beta^{(l)}||_{2}+\alpha||\beta||_{1}\right\},\end{align*}

where $l(\beta )$ is a loss function that depends on the phenotype information, $n$ represents the number of samples, $\lambda>0$ and $0\leq \alpha \leq 1$ are regularization parameters, $\beta $ is the regression coefficient vector and $\beta ^{(l)}$ is its subvector corresponding to the $l$th group. If the phenotype information on cancer diagnosis (or treatment response) is utilized, then sample label $y_{i}$ is encoded as 1 or 0, and the negative log-likelihood function is adopted 


(3) \begin{align*}& l(\beta)=\sum_{i=1}^{n}\sum_{l=1}^{s}(\log(1+\exp(x_{i}^{(l)T}\beta^{(l)}))-y_{i}x_{i}^{(l)T}\beta^{(l)}).\end{align*}

If the phenotype information on survival is utilized, then the following loss function is adopted: 


(4) \begin{align*}& l(\beta)=\sum_{l=1}^{s}\log \left(\sum_{i\in D} \left(\sum_{j\in R_{i}}\exp \left(x_j^{(l)T}\beta^{(l)}\right)-x_i^{(l)T}\beta^{(l)}\right)\right)\end{align*}

where $D$ is the failure index set of samples determined by the occurrence of events, and $R_{i}$ is the index set of samples with survival time longer than that of the $i$th sample.

The $\beta $ in (2) can be solved through the R package ‘SGL’. The regression coefficient reflects the cell’s impact on the phenotype, with positive and negative coefficients indicating associations with higher and lower value-encoding phenotypes, respectively. In cases where the phenotype represents survival information, positive coefficients correspond to cells that are consistently associated with worse survival outcomes. To simplify, we denoted cells as LP_SGL+ cells (positive coefficients), LP_SGL- cells (negative coefficients) and Background cells (coefficients equal to 0).

During the implementation of the LP_SGL model, three parameters need to be determined: the resolution parameter $\gamma $, regularization parameters $\alpha $ and $\lambda $. The $\gamma $ acts as a threshold, requiring a minimum density of $\gamma $ within each group. Higher values of $\gamma $ result in more groups being obtained. We used a sequence of $\{0.3, 0.6, 0.9, 1.2, 1.5, 1.8\}$ to test the impact of different $\gamma $ values on the results, with detailed results presented in Supplementary Table 1 (see Supplementary Data available online at https://academic.oup.com/bib). Due to minimal fluctuations in the results as $\gamma $ changed for each dataset, we simplified the experimental process by setting $\gamma $ to 0.6. The $\lambda $ determines the overall strength of the penalty term, while $\alpha $ balances the lasso and GL penalties. We created a search list of $\{0.005, 0.05, 0.1, 0.2, \cdots , 0.8, 0.9, 0.95\}$ in advance for $\alpha $. For each fixed $\alpha $, $\lambda $ was determined through 5-fold cross-validation, and the optimal parameter pair $(\alpha ^{*}, \lambda ^{*})$ was determined through experimental results.

### Datasets

The LUAD scRNA-seq data were downloaded from the ArrayExpress (accession numbers: E-MTAB-6149 and E-MTAB-6653), including 29 888 cells and 8 cell types [17]: cancer cell, endothelial cell, T cell, B cell, myeloid cell, alveolar cell, epithelial cell and fibroblasts cell. The bulk data of LUAD were downloaded from TCGA-LUAD. There are in total of 539 tumors and 59 normal samples, and 508 samples with overall survival time and status. An external bulk validation set of LUAD diagnosis was downloaded from GEO (accession code: GSE40419), including 87 tumors and 77 normal samples.

The melanoma scRNA-seq data (accession code: GSE115978) contained 6879 cells and 9 cell types [18]: T cell, CD4+ T cell, CD8+ T cell, B cell, macrophage, malignant cell, cancer-associated fibroblast (CAF), endothelial cell and Natural Killer (NK) cell. In reference [18], cells were defined as T cells based on the overall expression of established cell type markers (CD2, CD3D, CD3E, CD3G). T cells were further classified as CD8+ or CD4+ T cells if they expressed CD8 (CD8A or CD8B) or CD4, respectively, while the rest were still labeled as T cells. The melanoma bulk dataset PRJEB23709 was downloaded from [19]. There are in total of 46 treatment responders and 27 nonresponders. External bulk validation sets for melanoma and thymic carcinoma were downloaded from GEO (accession codes: GSE91061 and GSE181815, respectively).

The liver cancer scRNA-seq data (accession code: GSE125449) contained 8853 cells and 7 cell types [20]: CAF, tumor-associated macrophage (TAM), malignant cell, tumor-associated endothelial cell (TEC), cells with an unknown entity but express hepatic progenitor cell markers (HPC-like), T cell and B cell. TCGA-LIHC provides bulk data of 370 liver cancer samples with survival information, while GEO (GSE14520) provides another liver cancer bulk validation set with survival and recurrence information.

The gene expression values were averaged for genes with multiple occurrences of the same name during data preprocessing. For bulk data, a logarithmic transformation with a base of 2 was performed on the original count data. For scRNA-seq data, the R package ‘Seurat’ was used for preprocessing. Genes expressed in at least 400 cells were retained, and the filtered expression matrix was normalized using the ‘NormalizeData’ function. Highly variable genes between cells were identified using the ‘FindVariableFeatures’ function with the default ‘vst’ method. Subsequently, standardization and principal component analysis were performed using the ‘ScaleData’ and ‘RunPCA’ functions, respectively. The shared nearest neighbor graph was constructed based on the first 10 principal components using the ‘FindNeighbors’ function. Two-dimensional cell visualization was achieved using the ‘RunUMAP’ function.

### Testing and biological analysis

To assess the robustness of the model to incomplete or missing data, we deliberately removed some genes. We split the binary phenotype bulk data into two groups and used the R package ‘limma’ to identify DEGs between the two groups, based on the filtering criteria of Logarithm of fold change $|\log FC|$ greater than the threshold and *P*-value obtained by the default *t*-test less than 0.05. We set the threshold sequence as {0.5, 0.6, 0.7, 0.8, 0.9, 1} to obtain six different gene sets. The difference in gene sets resulted in different correlation matrices when integrating bulk data with scRNA-seq data. We evaluated the model’s robustness using six different incomplete datasets.

We conducted functional enrichment analysis on DEGs between LP_SGL+ cells and LP_SGL- cells. To assess the activity level of the over-expressed gene set across different samples, we employed the R package ‘GSVA’ to conduct gene set variation analysis (GSVA). We calculated a statistical test between the two types of samples using the *t*-test. Furthermore, we performed gene set enrichment analysis (GSEA) to investigate the enrichment of DEGs under different biological conditions. GSEA was implemented by utilizing the ‘gseGO’ and ‘gseKEGG’ functions in the R package ‘clusterProfiler’. *P*-values were calculated based on the hypergeometric distribution, and the false discovery rate (FDR) was calculated using the Benjamini–Hochberg method.

The lasso-cox model was implemented using the R package ‘glmnet’ based on DEGs between LP_SGL+ cells and LP_SGL- cells. Subsequently, multivariable Cox regression was performed using the R package ‘survival’ for genes with nonzero coefficients. Samples were then divided into high- and low-risk groups based on the median of predicted prognostic scores. To assess the difference in survival time between the two groups, Kaplan–Meier (K-M) survival analysis was conducted using the R package ‘survminer’, with the log-rank test. In addition, the Concordance index (C-index) was calculated to measure the predictive ability of the model. To avoid the contingency of the results, 10-times experiments were performed by setting seeds 1 to 10. For clinical characteristics-based methods including age, stage and sex, univariate cox regression was performed using the R package ‘survival’.

## RESULTS

### Identify cell subpopulations associated with LUAD and normal

**Figure 2:**
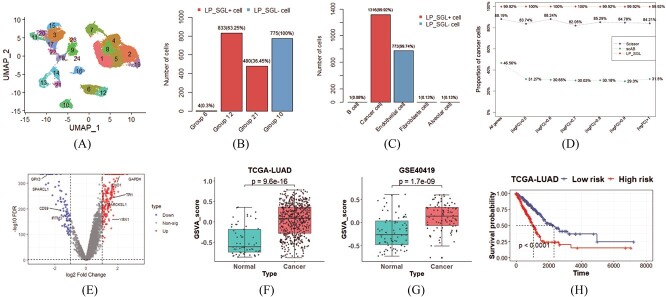
Experimental results on the LUAD dataset. (**A**) UMAP visualization of 24 cell groups obtained using the Leiden algorithm. (**B** and **C**) Bar chart of the distribution of LP_SGL+ cells and LP_SGL- cells with respect to cell groups and cell types, respectively. (**D**) Line chart of the proportions of cancer cells contained in the LUAD phenotype cells identified by LP_SGL, Scissor and scAB. (**E**) Volcano map of DEGs between LP_SGL+ cells and LP_SGL- cells. (**F** and **G**) Box plot of GSVA scores for cancer and normal samples on TCGA-LUAD and GSE40419 datasets, respectively. (**H**) K-M survival curves of high- and low-risk group samples divided by the median prognostic score in the TCGA-LUAD dataset.

We initially applied the LP_SGL method to LUAD dataset in order to identify cells that were associated with either the LUAD or normal phenotype. After preprocessing the data, 29 888 cells were assigned to 24 groups using the Leiden algorithm. The UMAP visualization of 24 cell groups and 8 cell types was, respectively, presented in Figure 2A and Supplementary Figure S1a (see Supplementary Data available online at https://academic.oup.com/bib). Subsequently, 1317 LP_SGL+ cells and 775 LP_SGL- cells were selected by implementing the LP_SGL. A bar chart of the distribution of LP_SGL+ cells and LP_SGL- cells with respect to cell groups was presented in Figure 2B and the corresponding UMAP visualization was displayed in Supplementary Figure S1b (see Supplementary Data available online at https://academic.oup.com/bib), and 63.25% (833/1317) and 36.45% (480/1317) of LP_SGL+ cells appeared in groups 12 and 21, respectively, while 100% of LP_SGL- cells were presented in group 10. A bar chart of the distribution of LP_SGL+ cells and LP_SGL- cells with respect to cell types was presented in Figure 2C and 99.92% (1316/1317) of LP_SGL+ cells were cancer cells and 99.74% (773/775) of LP_SGL- cells were endothelial cells. The concentrated characteristics observed in the distribution of LP_SGL+ cells and LP_SGL- cells within both cell groups and cell types demonstrated the ability of the LP_SGL to accurately identify phenotype-associated subpopulations by introducing cell group structure.

We then evaluated the robustness of LP_SGL, Scissor and scAB by using seven datasets (including six different incomplete datasets obtained under different thresholds). The line chart of the proportions of cancer cells contained in the LUAD phenotype cells identified by these methods was shown in Figure 2D. In the original data, the proportion of cancer cells contained in the LUAD-associated cells identified by LP_SGL was 99.92%, which was 11.73 and 53.36% higher than that identified by Scissor and scAB, respectively. On six incomplete datasets, the results obtained by LP_SGL remained almost unchanged, while the other two methods exhibited some degree of fluctuation.

To further reveal the biological significance of the identified cells, we performed differential expression analysis (DEA) between LP_SGL+ cells and LP_SGL- cells. A total of 210 upregulated and 89 downregulated genes were identified by setting $|\log FC|$ greater than 1 and the FDR less than 0.05. The volcanic plot of the DEGs was shown in Figure 2E. Notably, some of these genes have been identified as important regulatory factors in LUAD, such as *ENO1*, which has been previously reported to promote tumor progression in LUAD [21]. Similarly, *YBX1* has been shown to induce the migration of LUAD cells and contribute to tumor metastasis [22]. On the other hand, *GPX3* has been found to play an inhibitory role in LUAD, with lower expression levels in tumors compared with normal tissues [23]. These findings demonstrated the potential of LP_SGL for identifying significant DEGs that may be used as diagnostic or therapeutic targets for LUAD.

To assess the clinical relevance of the 210 over-expressed genes identified by LP_SGL, GSVA scores were calculated for each sample in bulk data. As shown in Figure 2F, the cancer samples exhibited significantly higher scores compared with the normal samples in the TCGA-LUAD dataset ($P=9.6e-16$). The same trend was observed in another independent LUAD dataset, as depicted in Figure 2G ($p=1.7e-09$). These results suggested that the identified upregulated genes were strongly correlated with LUAD. Furthermore, using survival information from the TCGA-LUAD dataset, 508 samples were divided into high- and low-risk groups based on the median predicted prognostic score. As presented in Figure 2H, the K-M survival curve indicated that samples with higher prognostic scores had significantly worse survival outcomes compared with those with lower scores. This analysis further supports the association of the identified LUAD-associated subpopulations with poor prognosis. As a result, we have successfully demonstrated the utility of LP_SGL in accurately identifying cell subpopulations associated with a particular phenotype.

### Identifying T cell subpopulations related to immunotherapy

**Figure 3:**
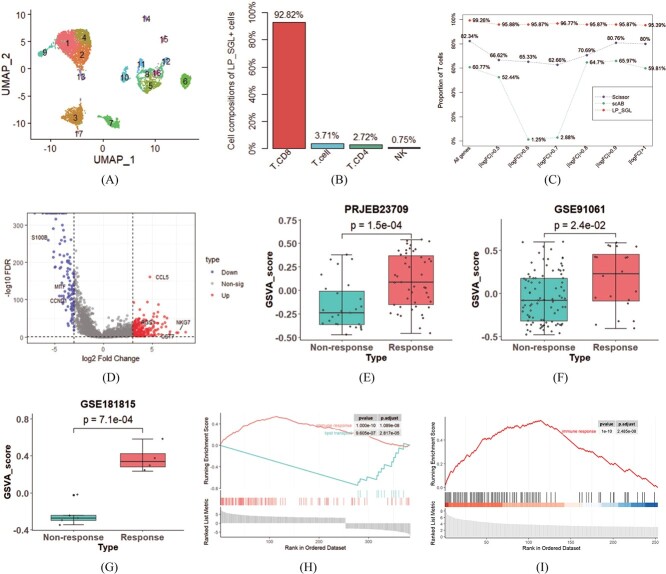
Experimental results on the melanoma dataset. (**A**) UMAP visualization of 17 cell groups obtained using the Leiden algorithm. (**B**) Bar chart of the distribution of LP_SGL+ cells with respect to cell types. (**C**) Line chart of the proportions of cancer cells contained in the response phenotype cells identified by LP_SGL,Scissor and scAB. (**D**) Volcano map of DEGs between LP_SGL+ cells and other cells. (**E**–**G**) Box plot of GSVA scores for response and non-response in PRJEB23709, GSE91061 and GSE181815 datasets, respectively. (**H**) GSEA plots of upregulated and downregulated biological processes (BP) of the overall DEGs. (**I**) GSEA plots of upregulated BP of the upregulated DEGs.

Understanding the mechanism behind the immune checkpoint blockade (ICB) response is crucial as it significantly improves the 10-year survival rate of melanoma patients, despite the therapy not benefiting most treated patients [24]. To address this issue, we employed LP_SGL to analyze melanoma data and identify T cell subpopulations associated with ICB response, and 6879 cells were assigned to 17 groups via the Leiden algorithm. The UMAP visualization of 17 cell groups and 9 cell types were, respectively, presented in Figure 3A and Supplementary Figure S2a (see Supplementary Data available online at https://academic.oup.com/bib). Then, 404 LP_SGL+ cells and 0 LP_SGL- cells were identified by implementing the LP_SGL. A bar chart of the distribution of LP_SGL+ cells with respect to cell types was presented in Figure 3B and the corresponding UMAP visualization was displayed in Supplementary Figure S2b (see Supplementary Data available online at https://academic.oup.com/bib). According to statistics, 99.26% (401/404) of LP_SGL+ cells were presented in group 1, showing the concentrated characteristic consistent with the experimental results on LUAD dataset. It is noteworthy that 99.26% (401/404) of LP_SGL+ cells were T cells (CD8+ T cells: 92.82%, 375/404; CD4+ T cells: 2.72%, 11/404; T cells: 3.72%, 15/404), with the remaining 0.75% being NK cells. Recent research has highlighted the great potential of NK cells in cancer immunotherapy [25]. This result demonstrated that LP_SGL can accurately identify subpopulations related to ICB response, which has the potential to improve the effectiveness of immunotherapy for melanoma patients.

In addition, we tested the robustness of LP_SGL, Scissor and scAB by using seven datasets of melanoma. The line chart in Figure 3C showed the proportions of T cells contained in the response phenotype cells identified by these methods. In the original data, the proportion of T cells contained in the response phenotype cells identified by LP_SGL was 99.26%, which was 16.92 and 38.49% higher than that identified by Scissor and scAB, respectively. On six incomplete datasets, the results obtained by LP_SGL remained stable, while the other two methods exhibited significant fluctuations.

To gain a deeper understanding of the immunotherapy response mechanism, we performed DEA between LP_SGL+ cells and other cells, as LP_SGL- cells were not identified. A total of 253 upregulated and 131 downregulated DEGs were identified by meeting the criteria of $|\log FC|$ greater than 3 and FDR less than 0.05. The volcanic plot of the DEGs was shown in Figure 3D. Among them, many of these genes have been confirmed to be closely related to melanoma, such as the reduction of *MITF* level promoting melanoma invasion [26], tumor regression being abrogated by silencing *CCL5* [27] and *CST7* being significantly up-regulated in melanoma patients who respond to ICB treatment [28]. These results demonstrated that LP_SGL has the ability to identify gene signals related to immunotherapy responses.

We subsequently calculated the GSVA score of each sample to evaluate the clinical relevance of the identified DEGs. As shown in Figure 3E, the responder in the melanoma dataset had significantly higher scores than the nonresponder ($P = 1.5e-04$). Moreover, the external melanoma validation set showed similar results, as depicted in Figure 3F ($P = 2.4e-02$). Interestingly, we also tested whether the immunotherapy-associated cell subpopulations identified from melanoma dataset were applicable to thymic carcinoma samples. Surprisingly, as shown in Figure 3G, thymic carcinoma samples that responded to treatment had significantly higher scores than those that did not respond ($P = 7.1e-04$). Furthermore, the GSEA of the overall DEGs revealed overactivation of immune response processes and suppression of lipid transport processes, as shown in Figure 3H. The GSEA of the upregulated DEGs was shown in Figure 3I, while no significant enrichment of biological processes was observed for downregulated DEGs. These findings were consistent with previous research demonstrating that inhibiting lipid transport to melanoma cells effectively reduces their growth and invasion [29]. In summary, the LP_SGL identified cell subpopulations that were associated with ICB response, and the signal genes from these cells could reliably predict ICB response in melanoma and other types of cancer.

### Identifying cell subpopulations associated with worse survival in liver cancer

**Figure 4:**
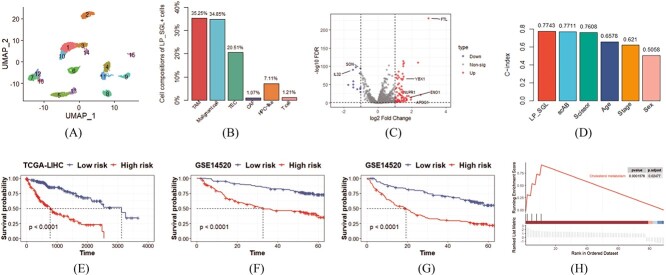
Experimental results on the liver cancer datasets. (**A**) UMAP visualization of 16 cell groups obtained using the Leiden algorithm. (**B**) Bar chart of the distribution of LP_SGL+ cells with respect to cell types. (**C**) Volcano map of DEGs between LP_SGL+ cells and LP_SGL- cells. (**D**) Bar chart of the average C-index of the results from 10-times experiments results. (**E**) The K-M survival curves of high- and low-risk group samples in the TCGA-LIHC dataset. (**F** and **G**) The survival and recurrence K-M curves of the high- and low-risk groups in the GSE14520 dataset, respectively. (**H**) Gene set enrichment analysis plots of upregulated Kyoto Encyclopedia of Genes and Genomes (KEGG) pathway of DEGs.

To further evaluate the model’s performance in survival phenotype data, we applied the LP_SGL method to the liver cancer dataset to identify cell subpopulations associated with poorer survival outcomes, and 8853 cells were assigned to 16 groups via the Leiden algorithm. The UMAP visualization of 16 cell groups and 7 cell types were, respectively, presented in Figure 4A and Supplementary Figure S3a (see Supplementary Data available online at https://academic.oup.com/bib), and 746 LP_SGL+ cells and 1243 LP_SGL- cells were identified. A bar chart of the distribution of LP_SGL+ cells with respect to cell types was presented in Figure 4B and the corresponding UMAP visualization was displayed in Supplementary Figure S3b (see Supplementary Data available online at https://academic.oup.com/bib), and 91.68% (684/746) of LP_SGL+ cells were composed of tumor-associated cells (TAM, CAF, TEC and malignant cell). Additionally, the cells identified by Scissor and scAB were labeled as Scissor+ cells, Scissor- cells and scAB+ cells, respectively, according to the habits of their respective papers. We applied scAB and Scissor to the liver cancer dataset and obtained the proportions of 85.14% (779/915) and 90.48% (19/21) tumor-associated cells in scAB+ cells and Scissor+ cells, respectively. LP_SGL identified a higher proportion of tumor-associated cells contained in cells associated with poorer survival phenotype compared with Scissor and scAB. Specifically, the proportion identified by LP_SGL was 1.2 and 6.54% higher than that identified by Scissor and scAB, respectively.

We conducted DEA between LP_SGL+ cells and LP_SGL- cells to explore potential biological mechanisms related to poorer survival. Figure 4C showed 77 upregulated and 12 downregulated DEGs that met the conditions of $|\log FC|$ greater than 1 and FDR less than 0.05. Among these DEGs, most of them have been reported to be associated with liver cancer, such as high expression of *YBX1* and *NUPR1*, which were associated with poor overall survival in liver cancer [30, 31]. In addition, overexpression of *IL32* has been found to inhibit cancer cell growth and may serve as a therapeutic target for various cancers, including liver cancer [32]. We also identified DEGs between scAB+ cells and other cells, as well as Scissor+ cells and Scissor- cells, using the same criteria. Subsequently, we used the DEGs obtained from each method to construct lasso-cox models. The average C-index of the 10-times experimental results corresponding to each method was presented in Figure 4D. We found that LP_SGL, scAB and Scissor all achieved comparable results and outperformed traditional clinical characteristic-based methods.

We conducted a survival analysis on the TCGA-LIHC dataset. As depicted in Figure 4E, there was a significant survival difference between the two groups, with the high-risk group having almost four times lower median survival time than the low-risk group. To verify the generalization of the identified DEGs, we conducted a survival analysis on an independent external validation set by following the same steps. The K-M survival curves of the high- and low-risk groups were shown in Figure 4F. We found that the high-risk group in the independent validation set still achieved worse survival outcomes. We also predicted the recurrence risk of the samples based on DEGs using the recurrence time and status of the samples. Figure 4G showed a significant difference in recurrence between the high- and low-risk groups. Furthermore, we performed GSEA based on DEGs and found that the cholesterol metabolism pathway was significantly enriched (Figure 4H). As the liver is the main organ responsible for cholesterol metabolism, abnormal cholesterol metabolism has been associated with the occurrence of liver diseases [33].

## DISCUSSION

In this paper, we proposed LP_SGL to identify phenotype-associated subpopulations by integrating scRNA-seq, bulk expression and bulk phenotype data. Importantly, our method was applicable to binary, survival and linear phenotype data, although we were unable to demonstrate the linear experiment due to a lack of suitable data. Moreover, our method can be extended to other omics data, such as chromatin accessibility and DNA methylation data. We also evaluated the performance of cell grouping using the Louvain algorithm (Supplementary Table 2, see Supplementary Data available online at https://academic.oup.com/bib), and the comparable results indicated that incorporating cell group structure into the model was effective. This provides a new perspective for incorporating other cell clustering methods into integrated multi-omics data models.

We compared the proposed LP_SGL with the currently mainstream phenotype-associated subpopulation identification methods, Scissor [9] and scAB [10], where the data preprocessing and parameter settings of both methods were consistent with their respective original literature. The LP_SGL selected the highest proportions of cancer cells and T cells when the three methods were applied to the LUAD diagnosis, melanoma drug response and liver cancer survival datasets, respectively. It is worth noting that compared with LP_SGL and Scissor, scAB consistently selects the highest number of cells, which may be the reason why the cells it identifies contain a lower proportion of cancer cells or T cells. The LP_SGL selected a larger number of cells than Scissor on both LUAD and liver cancer datasets. On the melanoma dataset, LP_SGL identified 404 LP_SGL+ cells in the optimal results. Moreover, when LP_SGL identified 1406 LP_SGL+ cells, which was more than the 1212 Scissor+ cells identified by Scissor, the proportion of T cells in LP_SGL+ cells was 95.87%, still higher than its proportion in Scissor+ cells. These results indicated that LP_SGL had a more accurate and comprehensive ability to identify phenotype-associated subpopulations.

Flow cytometry is a prevalent technique in experiments for identifying cell subpopulations [34]. It enables the segregation of target cells from a mixed cell population based on the fluorescence signal of cell surface markers [35]. However, since our research primarily focused on exploring phenotype-associated subpopulations using available transcriptomic data, there is currently no available flow cytometry data for identifying cell subpopulations. In ensuing studies, integrating flow cytometry data with our algorithm will be on our agenda. Moreover, the patients who underwent bulk RNA-seq in this study are different from those who underwent scRNA-seq. This rendered us incapable of scrutinizing the distribution of identified cells in response and nonresponse samples. Nevertheless, the comparison of performance among LP_SGL, Scissor and scAB, along with extensive biological analyses, proved the credibility of the proposed LP_SGL. Utilizing data from patients who have undergone both bulk RNA-seq and scRNA-seq may be advantageous in identifying phenotype-associated subpopulations. This will be a focus of our future research.

**Key Points**

- Our proposed method LP_SGL for integrating scRNA-seq, bulk expression and bulk phenotype data.
- The group effects caused by cell–cell interactions were introduced into the model to guide the identification of phenotype-associated subpopulations.
- LP_SGL identified a higher percentage of cancer cells, T cells and tumor-associated cells than Scissor and scAB on lung adenocarcinoma diagnosis, melanoma drug response and liver cancer survival datasets, respectively.
- The biological analysis on three original datasets and four independent external validation sets demonstrated that the signaling genes of this cell subset have the ability to predict cancer, immunotherapy and survival.

## Supplementary Material

supplementary_material_for_lp_sgl_bbad424

## Data Availability

Codes for LP_SGL are freely available in the GitHub repository (https://github.com/hongmeizhanghm/LP_SGL). The data underlying this article are available in the article.

## Author Biographies

**Juntao Li** is a full professor at the College of Mathematics and Information Science, Henan Normal University. His research interests include machine learning and data mining.

**Hongmei Zhang** is a master student at the College of Mathematics and Information Science, Henan Normal University. Her research include machine learning and RNA-seq data analysis.

**Bingyu Mu** is a lecturer at the School of the Art and Design, Zhengzhou University of Light Industry. Her research include interface and interaction, virtual reality and digital twin.

**Hongliang Zuo** is a full professor at the College of Mathematics and Information Science, Henan Normal University. His research interests include machine learning and pattern recognition.

**Kanglei Zhou** is currently pursuing a Ph.D. at the School of Computer Science and Engineering, Beihang University. His research include human motion analysis and augmented reality.
