# Supplementary material for: Identifying phenotype-associated subpopulations through LP_SGL
Source: Brief Bioinform. 2023 Nov 25;25(1):bbad424. doi: 10.1093/bib/bbad424 (PMC10753413; doi:10.1093/bib/bbad424)
Supplement: supplementary_material_for_lp_sgl_bbad424 [file supplementary_material_for_lp_sgl_bbad424.doc]

**SUPPLEMENTARY INFORMATION**

**Identifying phenotype-associated subpopulations through LP_SGL**

**Juntao Li^1^ , Hongmei Zhang,^1,∗^ Bingyu Mu,^2,∗^ Hongliang Zuo^1^ and Kanglei Zhou^3^**

1 College of Mathematics and Information Science, Henan Normal University, 46 Jianshe East Road, 453007, Xinxiang, China

2 College of Arts and Design, Zhengzhou University of Light Industry, No. 5 Dongfeng Road, 450000, Zhengzhou, China

3 School of Computer Science and Engneering, Beihang University, 37 Xueyuan Road, Haidian District, 100191, Beijing, China

* Correspondence: zhanghmmail@163.com, dianesaidmok@gmail.com

**Supplementary Figures**


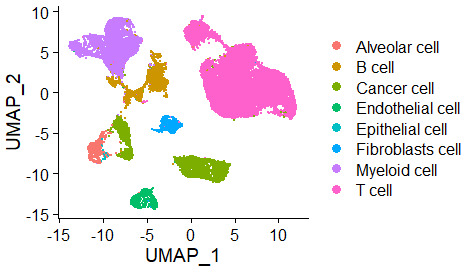

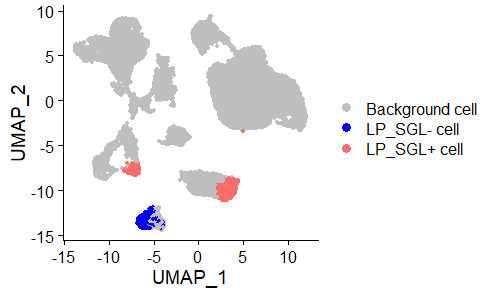


(a) (b)

**Supplementary Fig. S1.** Experimental results on the LUAD dataset. (a) UMAP visualization of 8 cell types.

(b) UMAP visualization of the distribution of LP_SGL+ cells and LP_SGL- cells.


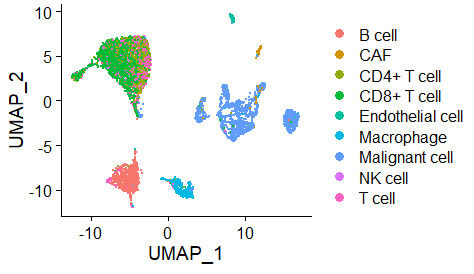

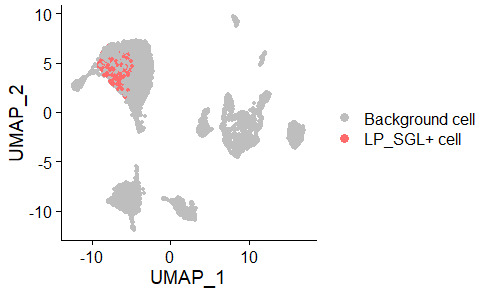


(a) (b)

**Supplementary Fig. S2.** Experimental results on the melanoma dataset. (a) UMAP visualization of 9 cell types.

(b) UMAP visualization of the distribution of LP_SGL+ cells.


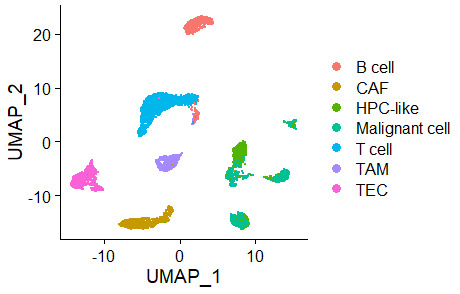

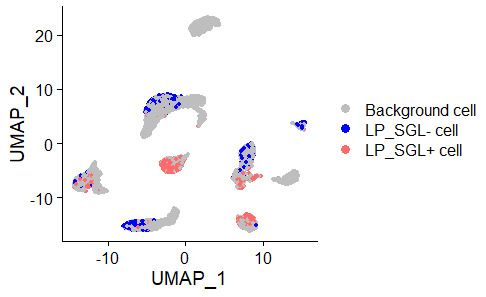


(a) (b)

**Supplementary Fig. S3.** Experimental results on the liver cancer dataset. (a) UMAP visualization of 7 cell types. (b) UMAP visualization of the distribution of LP_SGL+ cells and LP_SGL- cells.

**Supplementary Tables**

Supplementary Table 1. The proportion of cancer cells, T cells, and tumor-associated cells contained in the LP_SGL+ cells obtained by LP_SGL on LUAD, melanoma, and liver cancer datasets when different γ values were set.

| Datasets | γ=0.3 | γ=0.6 | γ=0.9 | γ=1.2 | γ=1.5 | γ=1.8 |
| --- | --- | --- | --- | --- | --- | --- |
| LUAD | 99.78% | 99.92% | 99.93% | 99.71% | 100% | 100% |
| Melanoma | 99.00% | 99.26% | 99.23% | 99.04% | 98.13% | 98.46% |
| Liver cancer | 89.39% | 91.69% | 93.20% | 91.00% | 91.69% | 90.91% |

Supplementary Table 2. The proportion of cancer cells, T cells, and tumor-associated cells contained in LP_SGL+ cells obtained by LP_SGL (Louvain algorithm) on LUAD, melanoma, and liver cancer datasets.

| Datasets | All genes | \|logFC\|>0.5 | \|logFC\|>0.6 | \|logFC\|>0.7 | \|logFC\|>0.8 | \|logFC\|>0.9 | \|logFC\|>1 |
| --- | --- | --- | --- | --- | --- | --- | --- |
| LUAD | 99.92% | 100% | 100% | 100% | 99.92% | 99.92% | 99.92% |
| Melanoma | 99.27% | 95.87% | 95.87% | 96.96% | 95.87% | 96.03% | 95.40% |
| Liver cancer | 91.95% | - | - | - | - | - | - |
